# Fluctuating Behavior and Influential Factors in the Performance of the QuantiFERON-TB Gold In-Tube Assay in the Diagnosis of Tuberculosis

**DOI:** 10.1371/journal.pone.0103763

**Published:** 2015-08-19

**Authors:** Lei Bao, Tao Li, Ni Diao, Yaojie Shen, Lingyun Shao, Ying Zhang, Shuihua Lu, Wenhong Zhang

**Affiliations:** 1 Department of Infectious Diseases, Huashan Hospital, Fudan University, Shanghai, China; 2 Department of Tuberculosis Disease, Shanghai Public Health Clinical Center, Fudan University, Shanghai, China; 3 Department of Molecular Microbiology and Immunology, Bloomberg School of Public Health, Johns Hopkins University, Baltimore, Maryland, United States of America; 4 Institutes of Biomedical Sciences, Fudan University, Shanghai, China; Glaxo Smith Kline, DENMARK

## Abstract

**Background:**

The QuantiFERON-TB Gold In-Tube (QFT-GIT) is a newly developed but widely used interferon-γ release assay for diagnosing tuberculosis (TB). However, research has not determined whether age or the use of an immune suppressive or anti-TB treatment influences this assay’s ability to detect TB. We assessed the QFT-GIT diagnostic performance for active tuberculosis (ATB) in children and adults in an endemic country and explored the effects of glucocorticoids and anti-TB therapy on the diagnostic value of the QFT-GIT.

**Methods:**

A total of 60 children and 212 adults with suspected ATB were evaluated with the QFT-GIT. The association between the QFT-GIT diagnostic value and pretreatment factors was qualitatively and quantitatively assessed.

**Results:**

The sensitivity of the QFT-GIT was 83.9% (95% CI 66.3%-94.6%) in children, and 73.7% (95% CI 57.8%-85.2%) in adults. Glucocorticoids affected the mitogen-stimulated response in both children and adults. In subjects undergoing glucocorticoid pretreatment, 25.0% of the children presented with false-negative QFT-GIT results, 28.6% of adults presented with indeterminate results. For subjects pre-treated with anti-TB drugs, 44.4% presented with false-negative QFT-GIT results.

**Conclusions:**

The QFT-GIT has higher sensitivity and specificity in children than adults. Glucocorticoid treatment negatively impacts the diagnostic value of the QFT-GIT in all age groups. Anti-TB treatment decreases the sensitivity of the QFT-GIT. Therefore, we recommend that the QFT-GIT assay be performed before TB-specific treatment is initiated and the test should not be used on people undergoing immunosuppression treatment, regardless of their age. A quantitative analysis of the QFT-GIT could be useful for assessing and monitoring TB-specific and non-specific immunity during conversion of the disease.

## Introduction

Tuberculosis (TB) remains a serious public health threat. According to the latest World Health Organization report, there were 8.6 million new TB cases and 1.3 million deaths in 2012 [1]. Childhood TB has become more prevalent, with 490,000 new childhood cases of TB and 64,000 deaths reported in 2011 [2]. In all age groups, global prevalence of TB illustrates the importance of TB diagnosis and surveillance, especially for countries that have a high burden of TB, such as China.

However, the high use of the Bacillus Calmette-Guerin (BCG) vaccination and the prevalence of latent TB infection (LTBI) can interfere with accurately diagnosing active TB (ATB). There are currently no optimal diagnostic criteria for ATB; each diagnostic method has limitations. The various clinical symptoms for ATB can lead to atypical chest X-ray presentations [3]. Bacterial cultures must be incubated for at least two weeks before they yield results, and an estimated 20% of all TB patients have negative sputum-smear and culture tests [4]. The tuberculin skin test (TST) has a low specificity in the BCG-vaccinated population due to the cross-reactivity of the diagnostic antigens that are used in the TST for BCG vaccine strains [5, 6]. Additional, collecting biopsy tissues is traumatic. Xpert MTB/RIF is a molecular diagnostic test which has been approved by WHO and are applied in many countries in the detecting of TB and rifampin-resistant TB [1]. Studies have shown that sensitivity of this test was high in smear-positive patients [7]. However, the sensitivity was much lower in smear negative patients or patients with non-respiratory samples [7, 8]. Therefore, new diagnostic methods for TB are urgently needed.

T-cell-based IFN-γ release assays (IGRAs) have recently been developed. The two commercially available IGRA approaches are the whole blood-based QuantiFERON-TB Gold In-Tube (QFT-GIT) and the peripheral blood mononuclear cell (PBMC)-based T-SPOT.TB assay. ELISA (enzyme-linked immunosorbent assay) or ELISPOT (enzyme-linked immunospot assay) are used in IGRAs to detect *Mycobacterium tuberculosis* (MTB)-specific antigen-stimulated IFN-γ production. MTB-specific antigens early secreted antigenic target 6 (ESAT-6) and culture filtrate protein 10 (CFP-10), both of which are encoded by the RD1 (region of difference 1) of MTB, are absent from BCG strains and most non-TB mycobacteria, so the specificity of IGRAs for detecting MTB infections is superior to that of the TST [9, 10].

While there have been numerous studies performed in adults, few studies have been conducted on the use of IGRAs in children. In an early study conducted in Germany, a country that stopped using BCG vaccination in 1998, the authors found that QFT-GIT sensitivity for diagnosing childhood TB disease is as high as 93% (95% CI, 77%-99%) [3]. The authors of one review reported that QFT-GIT sensitivity for diagnosing ATB ranges from 62% to 94% [10]. To investigate the diagnostic value of the QFT-GIT, we compared the performance of the QFT-GIT in consecutive hospitalized children and adults with suspected ATB. To evaluate the factors that influence the diagnostic value of the QFT-GIT, we assessed the effects of common clinical factors, such as the use of glucocorticoids and anti-TB therapies, on the performance of the QFT-GIT.

## Materials and Methods

### Study population

A total of 272 subjects with clinically suspected ATB were enrolled in this study, including 60 children younger than 16 years of age who were admitted to the Shanghai Public Health Clinical Center and Children’s Hospital of Fudan University from December 2010 to August 2011, and 212 adults who were admitted to the Huashan Hospital of Fudan University between December 2010 and December 2011. The inclusion criteria are summarized in Table 1. Patients with confirmed ATB at enrollment, and patients with an indefinite diagnosis before analysis were excluded, as well as patients who were positive for HIV or who were treated in an outpatient setting. Patient demographic information, clinical symptoms, imaging presentations, pathological findings and treatment history were collected. The study was approved by the Huashan Institutional Review Board at Fudan University (approval number KY2009-309). All children were recruited after their guardians provided written informed consent, and all adult patients provided written informed consent.

**Table 1:** Inclusion and exclusion criteria for the study population.

**Inclusion Criteria**	
	One or more of the following criteria:
	1. T of more than 37.5°C, with or without weakness and night sweat.
	2. Cough, expectoration, hemoptysis or chest pain.
	3. Abdominal distension, abdominal pain, diarrhea, or hematochezia.
	4. Frequent micturition, urgent micturation, odynuria or hemauria.
	5. Headache, vomiting or unconsciousness.
	6. Arthralgia, lumbar and back pain, with or without pathological fractures, and movement disorders.
	7. Rash, subcutaneous mass, or lymphadenectasis.
**Exclusion Criteria:**	
	1. HIV test positive.
	2. Already confirmed active tuberculosis
	3. Outpatients with an indefinite final diagnosis because of transfers to outpatient treatment.

### Definition and classification

All subjects with suspected ATB at enrollment were classified into the following four groups according to their diagnosis after enrollment: 1) confirmed TB; 2) highly probable TB; 3) possible TB; 4) not TB [11]. The category definitions are summarized in Table 2. The diagnosing clinicians were blind to the QFT-GIT outcome.

**Table 2:** Diagnostic classification of the patients.

Clinical Diagnostic Groups	Definition of the Case Categories
**Confirmed tuberculosis**	Patients with clinical specimens (e.g., sputum, urine, cerebrospinal fluid, pus and biopsy tissue) that are positive for mycobacterium tuberculosis in culture, acid-fast stain or PCR.
**Highly probable tuberculosis**	Patients with at least one of the following symptoms:
	1. Chest X-ray presentations suspected for active tuberculosis:
	a. Miliary findings or tuberculoma in the lung.
	b. Nonpyogenic pleural effusion.
	c. Cavitation associated with subacute or chronic pneumonia.
	2. Caseating granuloma in histology or biopsy tissue.
	3. Diarrhea, hematochezia or ascites with abdominal lymphadenopathy.
	4. Destruction or compression fracture of the vertebral bodies or paravertebral abscess.
	5. Clinical improvement with anti-tuberculosis treatment.
**Possible tuberculosis**	Patients who do not qualify for the two aforementioned diagnoses for whom tuberculosis has not been ruled out.
**Not tuberculosis**	Clinical improvement without anti-tuberculosis treatment or definite evidence of other diseases

### QuantiFERON-TB Gold In-Tube

The QFT-GIT assay (Cellestis, Australia) was performed according to the manufacturer’s instructions. A nil control tube, TB antigen (ESAT-6, CFP-10, TB7.7) tube and mitogen tube (positive control) were used to collect whole blood from each subject (1ml/tube). All tubes were transferred to a 37°C incubator within 16 hours of collection. After a 16- to 24- hour incubation, the tubes were centrifuged, the plasma was removed and an ELISA was used to measure the IFN-γ (IU/mL) level. The level of IFN-γ release in response to MTB-specific antigens was calculated by subtracting the nil control tube from the TB antigen tube. The result was considered positive if the calculated result was at least 0.35 IU/mL and at least 25% more than the nil control value for any mitogen tube value. The result was considered negative if the positive control IFN-γ level minus nil control level was at least 0.5 IU/ml, and the calculated result was at least 0.35 IU/mL and less than 25% more than the nil control value, or no more than an additional 0.35 IU/ml. Indeterminate results were defined as those with a calculated result of less than 0.35 IU/ml, those with at least 0.35 IU/mL and less than 25% more than the nil control level and a positive control minus nil control value less than 0.5 IU/ml and those for which the nil tube value was more than 8.0 IU/ml. According to the instrument limitations, the upper limit of the test was 16 IU/ml and the lower limit was 0 IU/ml.

### Statistical Analysis

The sensitivity, specificity, positive predictive value (PPV) and negative predictive value (NPV) were calculated to evaluate the QFT-GIT assay. For sensitivity and specificity calculations, indeterminate results were included in the sum. The χ^2^ test was used to compare proportions, and the Mann Whitney test was used to compare the IFN-γ level in different groups. P-values of less than 0.05 were considered to be statistically significant. All data were analyzed with SPSS 19.0 and GraphPad Prism 5.

## Results

### Characteristics of the study subjects

**Fig 1:**
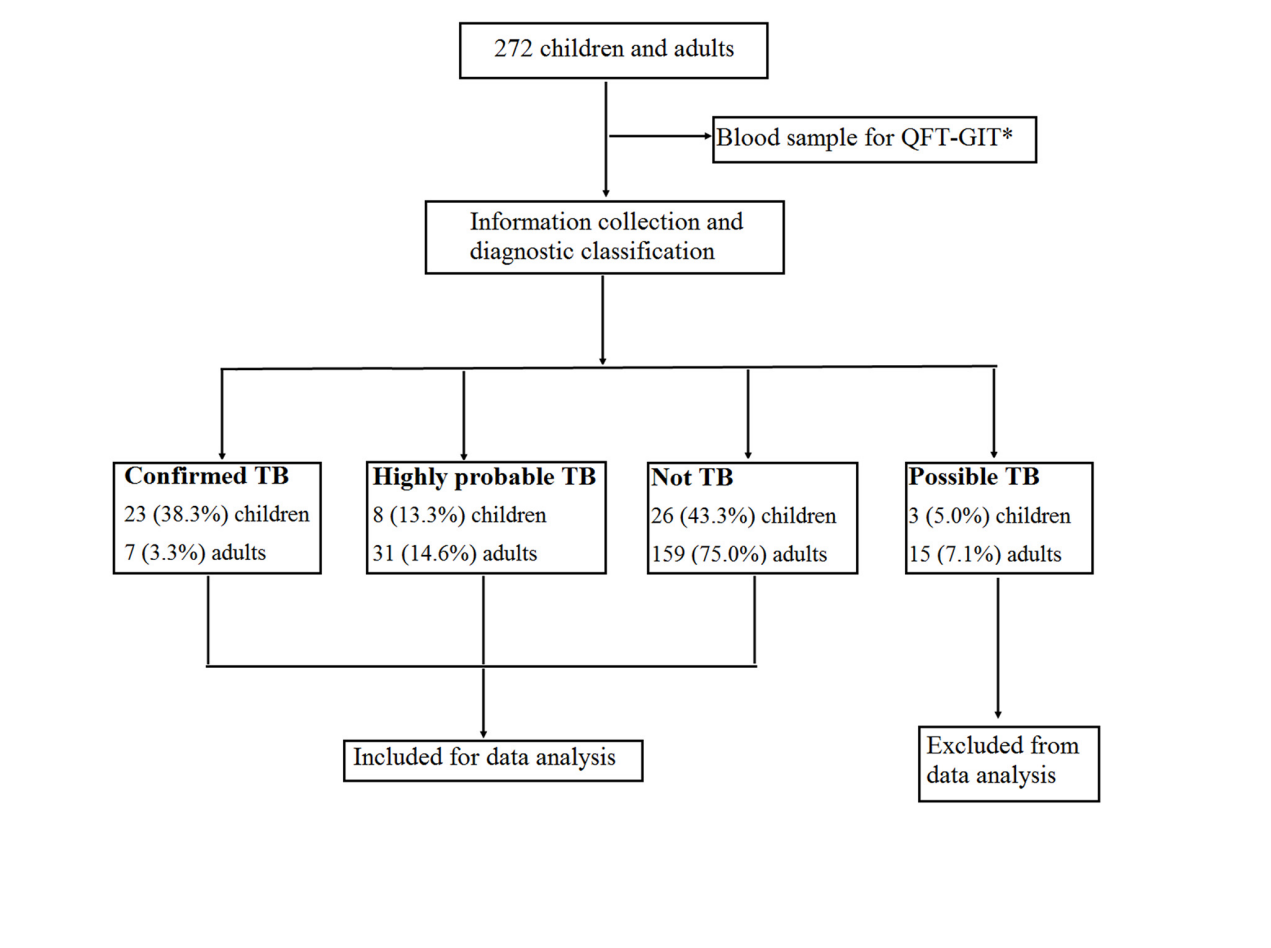
Recruitment and diagnostic classification of the participants. *QFT-GIT: QuantiFERON-TB Gold In-Tube.

The ‘possible TB’ group, including 3 children and 15 adults, was excluded from the data analysis (Fig 1). The demographic and clinical information of the remaining 3 groups are listed in Table 3. Thirty-one children and 38 adults were diagnosed with confirmed or highly probable TB, and 26 children and 159 adults were diagnosed with other diseases.

**Table 3:** Demographic and clinical details of the study subjects.

Clinical Feature	Confirmed TB		Highly probable TB		Not TB	
	Children	Adults	Children	Adults	Children	Adults
	N = 23	N = 7	N = 8	N = 31	N = 26	N = 159
**Mean age (children: months; adults: years)**	56	41.7	42.4	49.5	57.2	47.8
**Male (n, %)**	12 (52.2)	4 (57.1)	4 (50.0)	17 (54.8)	13 (50.0)	98 (61.6)
**Chest radiographs (n, %)**						
**Normal**	0 (0.0)	1 (14.3)	1 (12.5)	5 (16.1)	8 (30.8)	44 (27.7)
**Abnormal** ^★^	23 (100.0)	6 (85.7)	7 (87.5)	22 (71.0)	16 (61.5)	105 (66.0)
**Not performed/unavailable**	0 (0.0)	0 (0.0)	0 (0.0)	4 (12.9)	2 (7.7)	10 (6.3)
**Tuberculosis type (n, %)**						
**PTB† only**	5 (21.7)	1 (14.3)	4 (50.0)	4 (12.9)	0 (0.0)	0 (0.0)
**EPTB‡ only**	2 (8.7)	6 (85.7)	3 (37.5)	23 (74.2)	0 (0.0)	0 (0.0)
**Both PTB and EPTB**	16 (69.6)	0 (0.0)	1 (12.5)	4 (12.9)	0 (0.0)	0 (0.0)
**HIV-infected (n)**	0 (0.0)	0 (0.0)	0 (0.0)	0 (0.0)	0 (0.0)	0 (0.0)
**TB history**	0 (0.0)	0 (0.0)	0 (0.0)	1 (3.2)	0 (0.0)	16 (10.1)
**Close contact history**	5 (21.7)	1 (14.3)	2 (25.0)	2 (6.5)	0 (0.0)	7 (4.4)
**Glucocorticoid treatment(n,%)** *	11 (47.8)	3 (42.9)	1 (12.5)	11 (35.5)	0 (0.0)	67 (42.1)
**Anti-tuberculosis treatment(n,%)** **	0 (0.0)	2 (28.6)	0 (0.0)	14 (45.2)	0 (0.0)	14 (8.8)

★Abnormal chest radiograph included miliary findings, tuberculoma, pleural effusion, cavitation, inflammatory infiltrations, calcification, lymphadenectasis and other abnormal findings.

†PTB: pulmonary tuberculosis.

‡EPTB: extra-pulmonary tuberculosis.

* Glucocorticoids treatment within one month preceding the QFT-GIT assay. The dose was converted as prednisolone: at least 1 mg/kg/d for children and 5 mg/d for adults.

** Anti-tuberculosis treatment within three months preceding the QFT-GIT assay

### Performance of the QFT-GIT assay

The qualitative results of the QFT-GIT assay for three diagnostic groups are listed in Table 4. The positive test rates were comparable in the adults with confirmed and highly probable TB (71.4% vs. 74.2%), but the positive test rate was higher in children with confirmed TB than in children with highly probable TB (87.0% vs. 75.0%). For the sensitivity and specificity calculations, confirmed and highly probable TB cases were considered true ATB cases. The sensitivity (83.9% vs. 73.7%), specificity (88.5% vs. 70.4%) and PPV (92.9% vs. 47.5%) were all higher in children than in adults, respectively, and only the NPV (82.1% vs. 93.3%) was lower in children than in adults. In patients with only extrapulmonary TB (5 children and 29 adults), positive rate of QFT-GIT was 80% (4/5) in children and 65.5% (19/29) in adults. In comparison, in patients with only pulmonary TB (9 children and 5 adults), positive rate was 88.9% (8/9) in children and 100% (5/5) in adults. Additional diagnostic values for children and adults are summarized in Table 5.

**Table 4:** Qualitative result of the QFT-GIT for each diagnostic classification.

Test Result	Confirmed TB		Highly probable TB		Not TB	
	Children	Adults	Children	Adults	Children	Adults
	N = 23	N = 7	N = 8	N = 31	N = 26	N = 159
**Positive (%)**	20 (87.0)	5 (71.4)	6 (75.0)	23 (74.2)	2 (7.7)	31 (19.5)
**Negative (%)**	3 (13.0)	1 (14.3)	2 (25.0)	7 (22.6)	23 (88.5)	112 (70.4)
**Indeterminate (%)**	0 (0.0)	1 (14.3)	0 (0.0)	1 (3.2)	1 (3.8)	16 (10.1)

**Table 5:** The evaluation index for children and adults.

	Sensitivity	Specificity	PPV	NPV	Positive	Negative
	(%, 95% CI)	(%, 95% CI)	(%, 95% CI)	(%, 95% CI)	LR	LR
**Children**	83.9	88.5	92.9	82.1	7.3	0.2
	(66.3–94.6)	(70.2–96.8)	(76.5–99.1)	(63.1–93.9)		
**Adults**	73.7	70.4	47.5	93.3	2.5	0.4
	(57.8–85.2)	(62.9–77.0)	(35.3–60.0)	(87.2–96.8)		

PPV: Positive predictive value.

NPV: Negative predictive value.

LR: Likelihood ratio.

### The effects of glucocorticoids and anti-TB therapy on the QFT-GIT assay

**Fig 2:**
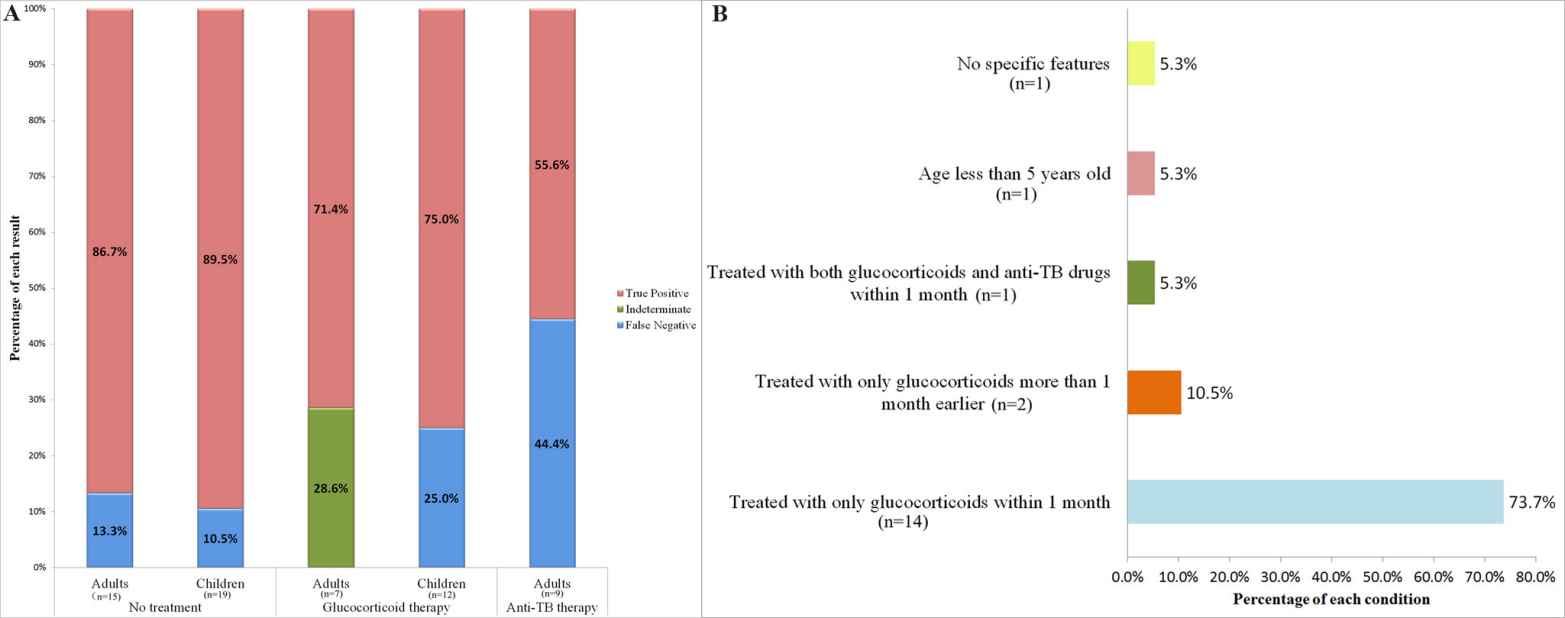
(A) The effects of glucocorticoids and anti-TB treatment on the QFT-GIT performance in children and adults with ATB. (B) The distribution of the indeterminate cases.

Of the patients with confirmed and highly probable TB, 38.7% (12/31) of the children and 36.8% (14/38) of the adults had been treated with glucocorticoids before the QFT-GIT test. Additionally, 16 adults (and no children) had received anti-TB therapy within 3 months before they were recruited; of these, 7 were also treated with glucocorticoids. To eliminate confounding factors in evaluating the effect of the two interventions on the assay performance, we removed the data from the 7 adults who underwent double interventions. Additionally, we stratified the remaining ATB patients into “no treatment”, “glucocorticoid therapy” and “anti-TB therapy” subgroups, and we recalculated the true positive and false negative rates for each subgroup. Compared to the “no treatment” subgroup, both therapy interventions had a negative effect on the test performance (Fig 2A).

There were a total of 19 indeterminate results. Of these, 73.7% (14/19) had therapy with glucocorticoids within the month preceding the QFT-GIT, 10.5% (2/19) were treated with glucocorticoids more than 1 month before the QFT-GIT, 5.3% (1/19) received both glucocorticoids and anti-TB treatment in the month preceding the QFT-GIT, 5.3% (1/19) were children, and 5.3% (1/19) lacked specific features (Fig 2B). Low IFN-γ release in response to mitogen stimulation was responsible for all 18 indeterminate adult results. T-SPOT.TB was tested for the indeterminate adults; the T-SPOT.TB test recognized 2 of 2 ATB patients, 15 of 16 non-TB patients and only one false positive case (Table 6).

**Table 6:** Results of the T-SPOT.TB for adults with indeterminate QFT-GIT results.

Patient	T-SPOT.TB	
	Positive	Negative
**Confirmed and highly probable TB (N = 2)**	2	0
**Not TB (N = 16)**	1	15

### Quantitative analysis of the QFT-GIT

**Fig 3:**
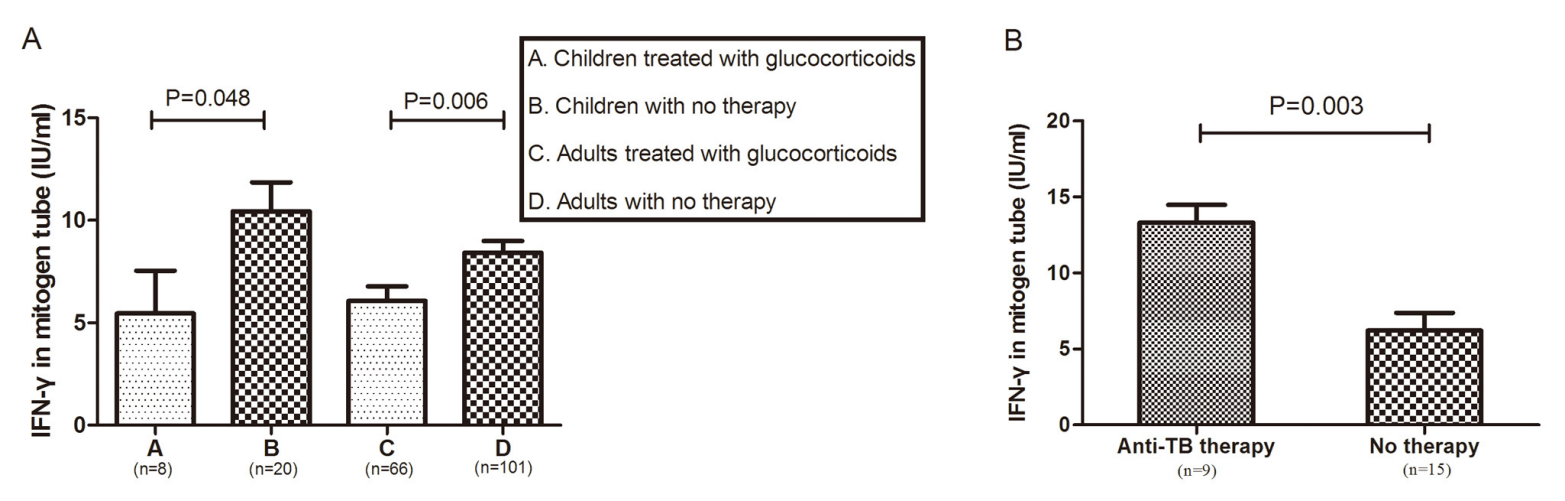
Comparison of the IFN-γ level in mitogen tubes in patients with different therapy regimens. (A) The effect of glucocorticoids on the mitogen-stimulated IFN-γ level between patients with and without ATB. ATB = active tuberculosis. non-ATB = diseases other than active tuberculosis. (Mean±SEM: column A: 5.45±2.09; column B: 10.44±1.41; column C: 6.06±0.71; column D: 8.42±0.58). (B) The effect of anti-TB therapy on IFN-γ in the mitogen tube in adults with ATB (Mean±SEM: Anti-TB therapy: 13.31±1.17; No therapy: 6.21±1.17).

**Fig 4:**
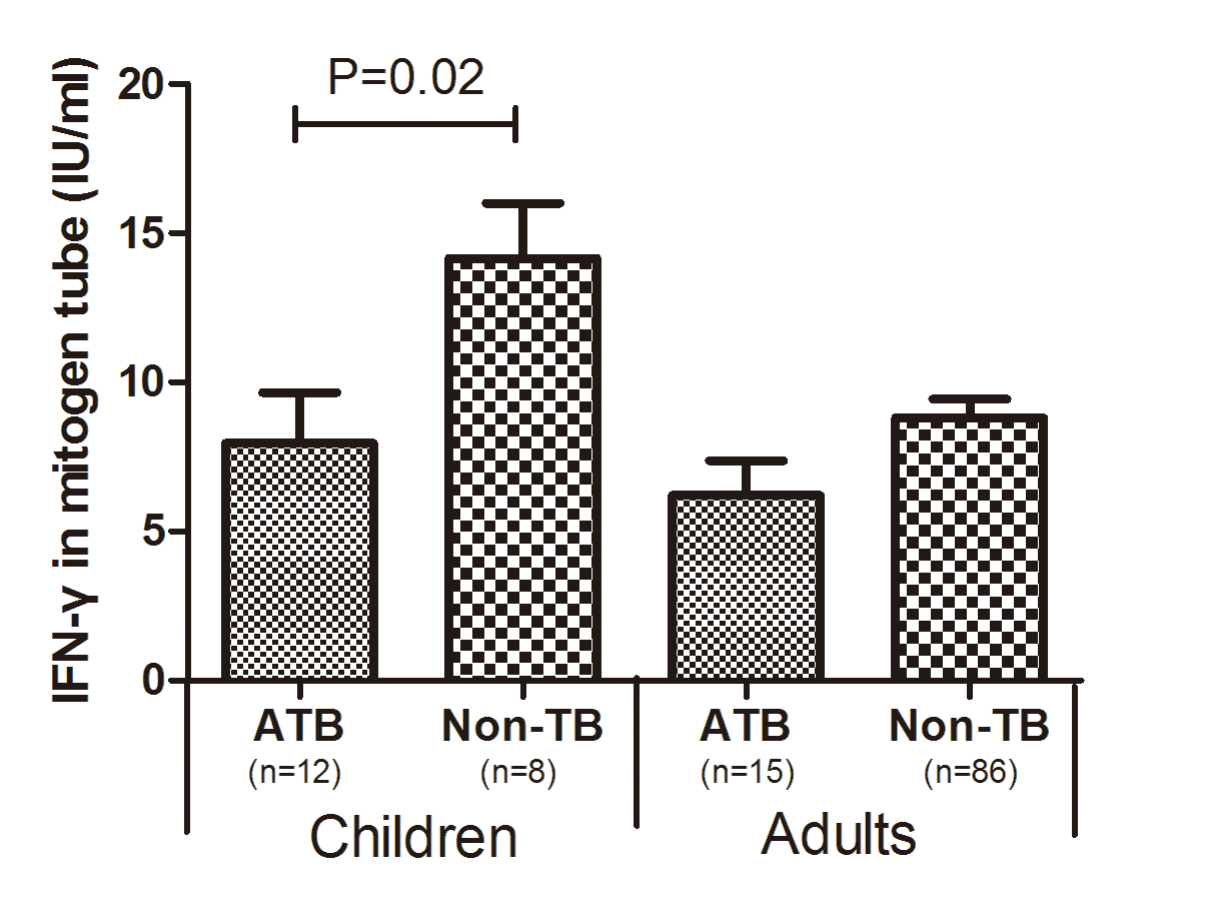
Comparison of mitogen-stimulated IFN-γ release between patients with and without ATB in the absence of treatment. (Mean±SEM: ATB children: 7.97±1.70; Non-TB children: 14.16±1.84; ATB adults: 6.21±1.17; Non-TB adults: 8.80±0.65).

We found a significant difference (P<0.05) in the mitogen tube IFN-γ levels between patients treated with and without glucocorticoids in both adults and children (Fig 3A). In the ATB cases, the average level of mitogen-stimulated IFN-γ release was 19.4% lower in adult patients treated with glucocorticoids than in the untreated patients (5.0 IU/ml vs. 6.2 IU/ml); the average mitogen-stimulated IFN-γ release was also 31.3% lower in children treated with glucocorticoids than in the untreated patients (5.5 IU/ml vs. 8.0 IU/ml). Glucocorticoid treatment also decreased the TB antigen-stimulated IFN-γ release by 6.5% in adults (4.3 IU/ml vs. 4.6 IU/ml) and 25.9% in children (4.0 IU/ml vs. 5.4 IU/ml). Anti-tuberculosis treatment increased the mitogen-stimulated lymphocyte response in ATB cases but not in non-ATB cases (Fig 3B). After eliminated the cases treated with glucocorticoids or anti-TB therapy, we found that the mitogen-stimulated IFN-γ level was lower in both children and adults with ATB than in non-ATB patients (Fig 4).

## Discussion

MTB and its components are potent inducers of monocyte cytokine production [12–14]. T lymphocytes activate and proliferate after antigens are processed and presented to them, and macrophage activation by the products of sensitized T lymphocytes, such as IFN-γ, constitutes TB-specific cell-mediated immunity (CMI). IGRAs can be used to examine IFN-γ release that is due to antigen-stimulated T lymphocyte activation. Our previous study revealed that anti-TB drugs have a negative conversion effect on IGRAs, which is consistent with the finding that approximately 38.5% of patients with ATB have negative T-SPOT.TB readings after 6 months of treatment with anti-TB drugs [15]. In this study, 44.4% of patients with ATB converted to a negative reading after less than 3 months of anti-TB treatment. The bactericidal effect on MTB reduces antigen production and subsequently reduces antigen-stimulated IFN-γ production. The best timing for performing IGRAs is before empirical treatment against MTB is initiated.

Various factors affect the performance of the QFT-GIT (Table 5; Fig 2A). Our finding that glucocorticoids lower the sensitivity of the QFT-GIT in both children and adults is consistent with previous studies [16–18]. Moreover, anti-TB treatment increases the false-negative rate, which decreases the test sensitivity in adults (83.9% vs. 73.7%). The higher sensitivity in children than in adults may be due to the higher rate of extrapulmonary TB patients and a higher percentage of patients receiving either glucocorticoids or anti-TB therapy in adults than in children. Positive rate was lower in adults with extrapulmonary TB (65.5%) and drugs can impact negatively on QFT-GIT. The comparative specificity between children and adults indicates a higher incidence of LTBI in the adult population. There was a much lower PPV in adults than in children (47.5% vs. 92.9%) for the following two reasons: the rate of LTBI was higher in adults than in children, and the incidence of ATB was lower in adults than in children (17.9% vs. 51.7%)., The ATB incidence may also cause a higher NPV in adults than in children (93.3% vs. 82.1%). In a high-burden setting, a recent study demonstrated that the T-SPOT.TB does not add incremental diagnostic value to other clinical and laboratory evidence for active TB in children [19]. Similarly, in our two cohorts, the positive likelihood ratios for children and adults were less than 10, and the negative likelihood ratios were greater than 0.1. This suggested that, regardless of age, the QFT-GIT did not increase the probability of confirming or ruling out ATB.

In the experiment, mitogen was used to activate all naïve and effector T cells in the tested blood. A significant decline in mitogen-stimulated IFN-γ release was found in both children and adults undergoing glucocorticoid therapy (Fig 3A), suggesting that glucocorticoids trigger non-specific suppression of T cell proliferation and activation. In ATB cases undergoing glucocorticoid therapy, mitogen-stimulated IFN-γ release decreased by 31.3% in children and 19.4% in adults. Simultaneously, the decline in the TB antigen-stimulated IFN-γ release was 25.9% in children and 6.5% in adults. Non-specific CMI defects may delay the establishment of TB-specific CMI in children. TB-specific CMI may be much stronger for adult patients than for children because adult patients have a higher risk of infection with MTB before developing ATB. Accordingly, TB-specific immunity may remain relatively active and may not be inhibited in a parallel with suppressed non-specific CMI.

After removing subjects treated with either interventions (glucocorticoids or anti-TB therapy) from the analysis, mitogen-stimulated IFN-γ release was lower in the ATB patients for both age groups (Fig 4). The direction of causality between immunosuppression and ATB merits discussion. On the one hand, insufficient non-specific CMI delays the establishment of TB-specific CMI or attenuates established TB-specific CMI, which leads to active disease. On the other hand, a downturn in CMI may result from the constantly consumption of circulating T cells in the clearance of MTB. During active disease, T lymphocytes are systemically mobilized, and the fluctuating range of mitogen-stimulated T cell activation in the bloodstream represents a dynamic interplay of T cell development, migration and consumption. When T cell consumption is faster than replenishment, the size of the “circulating T cell pool” shrinks. With increased bactericidal action of drugs, the reservoir of circulating T cells is gradually restored (Fig 3B). Because anti-TB drugs do not directly target a host’s CMI and subjects lacking ATB do not showed an elevated response after anti-TB therapy, the elevated mitogen-stimulated response may be a consequence of the lower effector T cell consumption in response to fewer microbial antigens.

Young age has been correlated with indeterminate QFT-GIT results in several studies [20–22]. However, of our 19 indeterminate cases, only one was a young patient (younger than 5 years old), and 90% of the cases were linked to glucocorticoid therapy (Fig 2B). Interestingly, glucocorticoid therapy does not seem to affect mitogen-stimulated IFN-γ release in the T-SPOT.TB assay as much as in the QFT-GIT (Table 6). The QFT-GIT is a whole blood IFN-γ release assay, and any changes in the function or the number of antigen-stimulated T cells would influence the QFT-GIT measurement of the total IFN-γ production. However, as for IGRAs, the T-SPOT.TB assay counts the number of antigen-specific T cells in 2.5X10^5^ of the PBMCs. Even in the presence of down-regulation in either the average activation level or the number of activated T lymphocytes, as long as the number of mitogen panel spots is more than 20, T-SPOT.TB does not yield indeterminate results. With the low mitogen-stimulated IFN-γ production in the QFT-GIT, our data suggests that even in the case of immunosuppression due to glucocorticoids or other unknown reasons, there are rarely fewer than 20 mitogen-stimulated T cells. The insensitivity of the T-SPOT.TB for detecting fluctuations in the proliferation and activation of PBMCs reduces indeterminate results, favoring interpretations for immunocompromised patients.

This study consecutively enrolled hospitalized patients; therefore, bias could result from the unbalanced number of patients and the non-matched clinical characteristics that were compared between groups, including age, immune status, and therapeutic regimen. The small sample size for each group may have biased the analysis factors influencing the QFT-GIT. The preliminary findings in this study will be further validated in future randomized controlled trials.

## Conclusions

Both the MTB load and the T lymphocyte function influenced the QFT-GIT diagnostic value. IGRAs should be performed as early as ATB is suspected because the diagnostic value of these tests deceases as soon as anti-TB drugs are administered. In immunocompromised individuals with a higher likelihood of indeterminate results, the T-SPOT.TB is an alternative to the QFT-GIT. According to our quantitative analysis, the QFT-GIT can detect non-specific and TB-specific CMI in the development and progression of TB.
